# Editorial on Special Issue “The Therapy of Alzheimer’s Disease: Towards a New Generation of Drugs”

**DOI:** 10.3390/pharmaceutics17030327

**Published:** 2025-03-03

**Authors:** Sílvia Chaves, M. Amélia Santos

**Affiliations:** Centro de Química Estrutural, Institute of Molecular Sciences, Departamento de Engenharia Química, Instituto Superior Técnico, Universidade de Lisboa, Av. Rovisco Pais 1, 1049-001 Lisboa, Portugal; silvia.chaves@tecnico.ulisboa.pt

## 1. Introduction

Alzheimer’s disease (AD) is the most prevalent age-dependent neurodegenerative disorder, leading to severe dementia. It is also one of the major causes of death worldwide. Despite enormous research efforts, no effective curative treatment options are available; thus, AD is one of the most urgent unmet medical needs. Only four drugs are globally approved (as of 2000): three cholinergic drugs (donepezil, rivastigmine, and galantamine) that mainly compensate for the loss of cholinergic neurons and one glutamatergic drug (memantine). However, these drugs can only temporarily address some symptoms. A second generation of drugs was recently (2021–2023) approved by the FDA, namely Aβ-directed monoclonal antibodies (aducanumab and lecanemab). However, aducanumab was later withdrawn from the market due to doubts about its efficacy and safety, while the efficacy of lecanemab is modest and it is exceedingly expensive (ca USD 27,000/patient/year). The absence of an effective drug has mainly been attributed to its recognized complex, though still not fully understood, multifactorial nature; therefore, the development of new affordable and effective treatments that can halt or slow down AD progression is still a major challenge in XXI century. In this regard, there is intensive research on new drugs that can address the multiple pathophysiological mechanisms involved in AD, including the development of multitarget-directed ligands (MTDLs) or pleiotropic drugs, to enable the simultaneous hitting of several of the most important targets associated with AD as opposed to the current single-target drug strategy.

This Special Issue provides an overview of the most recent developments, including innovative approaches, that are being followed to discover new small molecules with multitarget capacity as potential drugs to prevent, slow down, better manage, and ultimately cure the multifaceted and complex AD. Herein are included two review papers, namely **contribution 1**, which gives a comprehensive review of the recent conceptual strategies used in pleiotropic (MTDL) drug design, and **contribution 2**, which is focused on the two most important neurodegenerative diseases (NDs), AD and Parkinson’s disease (PD), summarizing the main hallmarks of these diseases, the available drugs, the groundbreaking research, as well as new directions that should be pursued for new disease-modifying drugs, namely based on polyfunctional approaches. The importance of exploring small MTDL drugs to combat AD’s multi-factorial pathogenesis is also illustrated in the three original scientific articles (**contributions 3–5**) that represent significant advances in developing innovative strategies to engineer new polyfunctional compounds combining several biological activities.

## 2. Overview of the Published Articles

The first review (**contribution 1**) provides a comprehensive overview, with some recent examples of AD therapies, of the different types of pleiotropic drugs (multitarget-directed ligands) that combine in the same molecular unit several activities directed against different targets, which are being studied or are already present on the market, with a focus on the structural aspect of drug design. Currently, the development of pleiotropic compounds is particularly attractive to medicinal chemists for the design of drugs aimed at the treatment of complex multifactorial diseases such as neurodegenerative diseases (e.g., AD) and cancers. These types of drugs should have clinical efficacy that is superior to that obtained with ordinary mono-target drugs used either individually or even in combination. In particular, if there is a synergy of the desired effects, it may be possible to reduce the active concentration administered, thus minimizing the undesirable side effects of ordinary active ingredients. The authors outlined the importance of this prerequisite in the concept of pleiotropic drugs, though they recognized the difficulties associated with demonstrating it for each target because of the laborious in vivo tests required. In particular, they made reference to examples of studies of the synergistic effects of administering the marketed drug Donepezil (AChE inhibitor) and a 5-HT4R agonist to mice. Other highlighted requirements are associated with the drug design, in particular, chemoinformatic tools for structure-based drug design and screening of chemical libraries to predict the successful capacity of a hybrid single molecule (as a non-dissociable or dissociable conjugate or even a prodrug) to reach the selected target and exhibit the proposed effect. The final but very important preliminary step is to analyze and select from among the different possibilities of chemically constructed molecular hybrid entities by incorporating (linking or merging) strategies while keeping all the structural requirements and compromises of previously defined ligand–target interactions.

Although they recognize the importance of these preliminary steps in the conceptual framework of pleiotropic drug design, the authors are aware of the fact that they require important and diverse interdisciplinary efforts, which may turn out to be a delicate process that is not always fully accomplished. However, whenever possible, to overcome the associated difficulties, the subsequent preclinical and clinical tests of the drug candidates will be facilitated, and the modest cost of preliminary drug design will help lower the life cycle of development of the drug candidate and the high costs of the later stages of clinical trials, thus justifying the current interest in the development of pleiotropic drugs in medicinal chemistry.

The second review in this special issue (**contribution 2**) is also focused on drug development for neurodegenerative diseases (NDs), bringing together groundbreaking research on both Alzheimer’s disease and Parkinson’s disease (PD); however, there is no breakthrough treatment available to date that can halt or reverse the progression of these complex multifactorial NDs. After summarizing the major hallmarks of AD and PD, the authors present a critical review of the current therapeutic approaches and the marketed drugs available for the pharmacological treatment of these diseases—mainly drugs approved by the American Food and Drug Administration (FDA). Recognizing that the treatments available for these NDs can only provide temporary symptomatic relief without halting or modifying disease progression, many researchers have followed different approaches over the last few decades. Most of these approaches are based on shifting from the single-target strategy of available drugs to multitarget-directed drug research aimed at unveiling the complex pathological mechanisms and identifying innovative disease-modifying drugs for AD and PD.

The authors also outline some potential directions that can be pursued to develop new effective drugs for treating AD and PD, including the control of downstream disease processes that can trigger neuroinflammation and protein misfolding, such as oxidative stress and metal dyshomeostasis. In this regard, the authors placed special emphasis on the importance of targeting and controlling the increased levels of oxidative stress markers and associated inflammatory processes that occur in the vulnerable brain and the central nervous system (CNS) of patients with these NDs. Another potential target of AD and PD that could be explored is adenosine receptors (ARs), a group of glycoproteins with several transmembrane domains that may be involved in the activation of a series of downstream signaling pathways. Although ARs are widely distributed in the human body and participate in a broad range of physiological and pathophysiological processes, some (A_1_Rs and A_2_Rs) are predominantly found in specific parts of the CNS. In particular, recent studies have revealed that high levels of specific A_2_Rs can lead to neuronal damage and PD progression, while others have also identified a close association between A_2_Rs and cognitive impairment in AD. Thus, the blockade of their activation using selective antagonists can be of therapeutic benefit to patients with these NDs, and some clinical trials— mostly for PD—are currently ongoing.

The work reported in **contribution 3** relates to the design, synthesis, and biological assessment of a series of 12 novel MTDLs. A special emphasis is given to the use of multicomponent reactions (MCRs), a synthetic tool that enables the generation of high structural diversity but involving environmentally friendly processes, in particular via one-pot, three-component Hantzsch reaction. The authors mainly use structure-based drug design by combining dihydropyridines (DHPs), which exhibit an inherent calcium channel modulation activity, and propargyl amide residues as bioactive segments included in several trade drugs due to their association with anti-stress oxidative pathways and potential inhibition of cysteine proteases such as Cathepsin S (Cat S), a facilitator of tau aggregation. Most of the developed MTDLs showed calcium channel antagonist activity and thus had potentially neuroprotective effects due to their inhibition of the development of Aβ peptides, and NFTs due to their DHP moiety. They also had antioxidant activity and ability to activate the endogenous Nrf2 antioxidant pathways due to the propargylamide moiety, although only two compounds demonstrated some capacity to inhibit Cat S.

The second original research article (**contribution 4**), although grounded on multitarget-directed ligands, also involves a drug-repositioning approach via molecular hybridization of the active principle moiety of the marketed drug (rivastigmine, RIV) with an indole (IND) moiety (as a surrogate of melatonin), resulting in one molecular entity with multitarget abilities. A series of nine rivastigmine–indole (RIV-IND) hybrids were designed, synthesized, and assayed for biological activity and radical scavenging capacity. Molecular docking studies revealed that all the compounds have dual enzyme (AChE, BChE) inhibitory capacity and, thus, a possibly higher disease-modifying impact than the mono-target (AChE) FDA-approved drugs. Some compounds presented higher AChE inhibition than the rivastigmine drug, while all the hybrids containing a hydroxyl substitution showed good radical scavenging ability and inhibition of Aβ_42_ self-aggregation, depending on the position of the hydroxyl group in the IND moiety. Cell viability and neuroprotection assays demonstrated that some compounds could rescue Aβ_1–42_- and iron/ascorbate-induced toxicity in the SH-SY5Y human neuroblastoma cell line. Finally, the in silico prediction of the drug-likeness of the hybrids showed that all the compounds seem to have potential oral availability. Overall, the authors expect that the results obtained for this novel class of RIV-IND hybrids will encourage further research on efficient multitarget drugs for AD therapy.

The third original research article (**contribution 5**) is also focuses on the design, synthesis, and biological assessment of new MTDL drugs. The research is mainly based on the modification of a lead MTDL rhein–huprine hybrid, which has recently been described by the authors of this contribution. This approach can also be considered a drug repositioning strategy since the former reported rhein–huprine hybrid is a combination of the pharmacophore huprine, a potent synthetic cholinergic compound (itself a hybrid of two cholinergic drugs, namely the natural drug Huperzine A and the synthetic drug Tacrine) and rhein, a hydroxyanthraquinone scaffold with putative tau anti-aggregating activity. These two large pharmacophores are connected by an oligomethylene chain, resulting in a very large molecular hybrid. Unfortunately, although this hybrid demonstrated good capacity to hit several targets with large binding sites, its large size compromises the drug metabolism and pharmacokinetic (DMPK) properties. Therefore, in this contribution, the authors reported a strategy used to reduce the molecular size of the rhein–huprine hybrid that is based on a stepwise structural modification of the complex tricyclic core of the dihydroxyanthraquinone moiety. The adopted stepwise strategy started with the removal of the hydroxyl substituents of the anthraquinone, followed by ring contracting, ring opening, and finally, ring elimination, resulting in a simple acetophenone moiety. Remarkably, after implementing these design strategies, the simplified acetophenone analog retained important multitarget capacity, i.e., potent inhibition of cholinesterases (AChE, BChE) and BACE-1, as well as Aβ and tau aggregation, while affording more favorable physicochemical and DMPK properties, thus confirming successful lead optimization via structural modification.

## 3. Conclusions and Future Directions

The overview of marketed therapies for AD (and PD), as well as the strategies used to find pioneering efficient drugs presented in this Special Issue, attempt to offer a glimpse into the state of the art. The proposed approaches are largely based on merging one molecular entity with several pharmacophoric entities, thus conferring multitarget ability, with or without drug repurposing, in order to overcome the drawbacks of the already approved mono-target drugs. Future development of anti-AD drugs will require various interdisciplinary efforts to maintain all the structural requirements and compromises for the already established ligand–target interactions and clinical approval, which will be a difficult task. In conclusion, the authors show that there are still immense possibilities for a combination of pharmacological effects for the future development of new disease-modifying drugs based on multitarget actions. We hope that the present intensive research will inspire the discovery of more efficient AD therapies, as well as new methods for earlier AD diagnosis. Unfortunately, a new generation of efficient clinical solutions may still have to wait another decade.

